# An X-ray beamline for utilizing intense, high-energy undulator radiation

**DOI:** 10.1107/S1600577525006903

**Published:** 2025-08-19

**Authors:** Hirokatsu Yumoto, Takahisa Koyama, Hiroshi Yamazaki, Yasunori Senba, Yujiro Hayashi, Taito Osaka, Ichiro Inoue, Kenji Tamasaku, Shunji Goto, Makina Yabashi, Haruhiko Ohashi

**Affiliations:** a Japan Synchrotron Radiation Research Institute 1-1-1 Kouto, Sayo-cho Sayo-gun Hyogo679-5198 Japan; b RIKEN SPring-8 Center, 1-1-1 Kouto, Sayo-cho, Sayo-gun, Hyogo679-5148, Japan; RIKEN SPring-8 Center, Japan

**Keywords:** high-energy X-rays, double multilayer monochromator, high-heat-load optics, X-ray optical instruments

## Abstract

The design, development and performance of an X-ray beamline for utilizing intense, high-energy undulator radiation are presented. A double multilayer monochromator, consisting of 150 pairs of 3.33 nm periodic Cr/C multilayers, provides intense X-rays by extracting a 1% energy bandwidth from high-order harmonic undulator radiation. We experimentally confirmed a high flux of 3.4 × 10^13^ (1.3 × 10^11^) photons s^−1^ with a beam size of 0.7 (0.3) mm (vertical) × 2.9 (∼4) mm (horizontal) at an X-ray energy of 100 (267) keV with a 1.0% (1.2%) energy bandwidth. Excellent performance of the high-energy high-flux X-ray beam was proven by conducting high-speed imaging analysis.

## Abstract 2

The design, development and performance of an X-ray beamline for utilizing intense, high-energy undulator radiation are presented.

## 1. Introduction

Fourth-generation large-scale synchrotron radiation sources, characterized with an extremely low emittance electron beam, enable the production of ultra-brilliant high-energy X-rays. These X-rays, which possess high penetrating power even for heavy metals, facilitate non-destructive and high-resolution visualization of the internal structure and properties, such as crystal grains, orientation and stress (Hayashi *et al.*, 2019 ▸), of large, dense and complex objects. Moreover, the coherence of these X-rays allows for the observation of the three-dimensional atomic structure within individual crystallites using Bragg coherent diffraction imaging (Richard *et al.*, 2023 ▸), elucidating the relationship between structure and function.

A particular distinction of fourth-generation sources from the third-generation sources is the reduction of the horizontal source size, achieving a point-like source instead of a line-shaped source. This characteristic contributes to the drastic improvement of the undulator spectrum given by
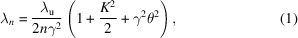
where λ_*n*_, λ_u_, γ, *K* and θ denote the wavelength of the harmonic *n*, the magnetic period length of the undulator, the relativistic Lorentz factor, the deflection parameter and the observation angle, respectively (Wiedemann, 2007 ▸). For third-generation sources, the spectrum of each order has a tail on the long wavelength side even for an on-axis aperture, because off-axis spectral components from the horizontally extended source are inevitably contaminated. In contrast, fourth-generation sources can produce a spectrum with a sharp peak for the on-axis aperture. A relative energy bandwidth at harmonic *n* is typically ∼1%, which is determined by the undulator period, the finite transverse electron emittance, and the energy spread of the electron beam. By extracting a single higher-order harmonic, one can utilize intense, high-energy X-rays with a moderate bandwidth of ∼1%, enhancing the photon flux in orders of magnitude, compared with that of Si crystal monochromators with a bandwidth below 0.01%. This intense ‘pink’ beam is suitable for various applications such as absorption contrast imaging, Compton scattering imaging, diffraction analysis and pair distribution function analysis.

The extraction of a single higher-order harmonic from the entire undulator spectrum is essential for utilizing intense and high-energy X-rays. One method involves the combination of a harmonic separator prism and a spatial aperture (Inoue *et al.*, 2018 ▸). In this method, the spectrum of the incident X-ray is spatially dispersed and the spatial aperture extracts an intended single higher-order harmonic. An alternative approach involves using a multilayer monochromator, which can extract a desired energy spectral bandwidth of ∼1%. The multilayer monochromator can isolate a single higher-order harmonic even at the current third-generation sources, enabling preliminary research of high-energy X-ray utilization at the next-generation facilities.

However, the development of multilayer monochromators for high-energy X-rays above several tens of keV is challenging. The substrate of a multilayer mirror, which generally has a larger grazing incidence angle than total reflection mirrors, must be finished with a higher accuracy, because the reflected wavefront is distorted in proportion to the grazing incidence angle. Even errors as small as 1 nm can significantly distort the reflected intensity distributions. Furthermore, a long substrate of several hundred millimetres in length is required to accept an incident beam with a width of ∼1 mm, due to a small grazing incident angle below several milliradians which is derived from Bragg’s law in the shorter wavelength range. This poses challenges in the fabrication of mirror substrates and multilayer coating. Meanwhile, very high power above 300 W and high-power density above 200 W mm^−2^ from the undulator radiation must be managed for stable use of optical components. In fact, only a few undulator beamlines supply high-energy X-rays above 50 keV from multilayer monochromators (Wilde *et al.*, 2016 ▸; Weiss *et al.*, 2019 ▸; Schropp *et al.*, 2020 ▸; Vaughan *et al.*, 2020 ▸; Schricker *et al.*, 2022 ▸).

This paper reports on an X-ray beamline designed to utilize intense, high-energy undulator radiation at 100 keV and beyond. A double multilayer monochromator (DMM) was developed to extract a single harmonic at a 1% bandwidth, and its performance was evaluated at the refurbished undulator beamline BL05XU of SPring-8, Japan. We provide an overview of the beamline design and optical components, with a focus on the DMM at 100 keV. We also demonstrate high-speed imaging using the resulting 100 keV beam.

## 2. Beamline design and instrumentation

### 2.1. Beamline overview

BL05XU began operations as an accelerator diagnostics beamline in 2004. In March 2017, the insertion device of this beamline was replaced from an out-of-vacuum undulator to an in-vacuum undulator (see the next section). In 2020, the beamline components in the optics hutches (OHs) were refurbished for R&D in high-energy X-ray optics and applications, as detailed below.

### 2.2. Beamline layout

#### 2.2.1. Light source

The undulator source of BL05XU is a device with 93 periods, a period length of 32 mm and a magnet length of 2.976 m. It generates horizontally polarized X-rays with a maximum magnetic field of 0.85 T and *K* of 2.54 at a gap of 8.24 mm. The maximum flux at 100 keV, used in subsequent DMM evaluations, is obtained using the 19th harmonic with *K* of 2.28.

#### 2.2.2. Components in optics hutches

**Figure 1:**
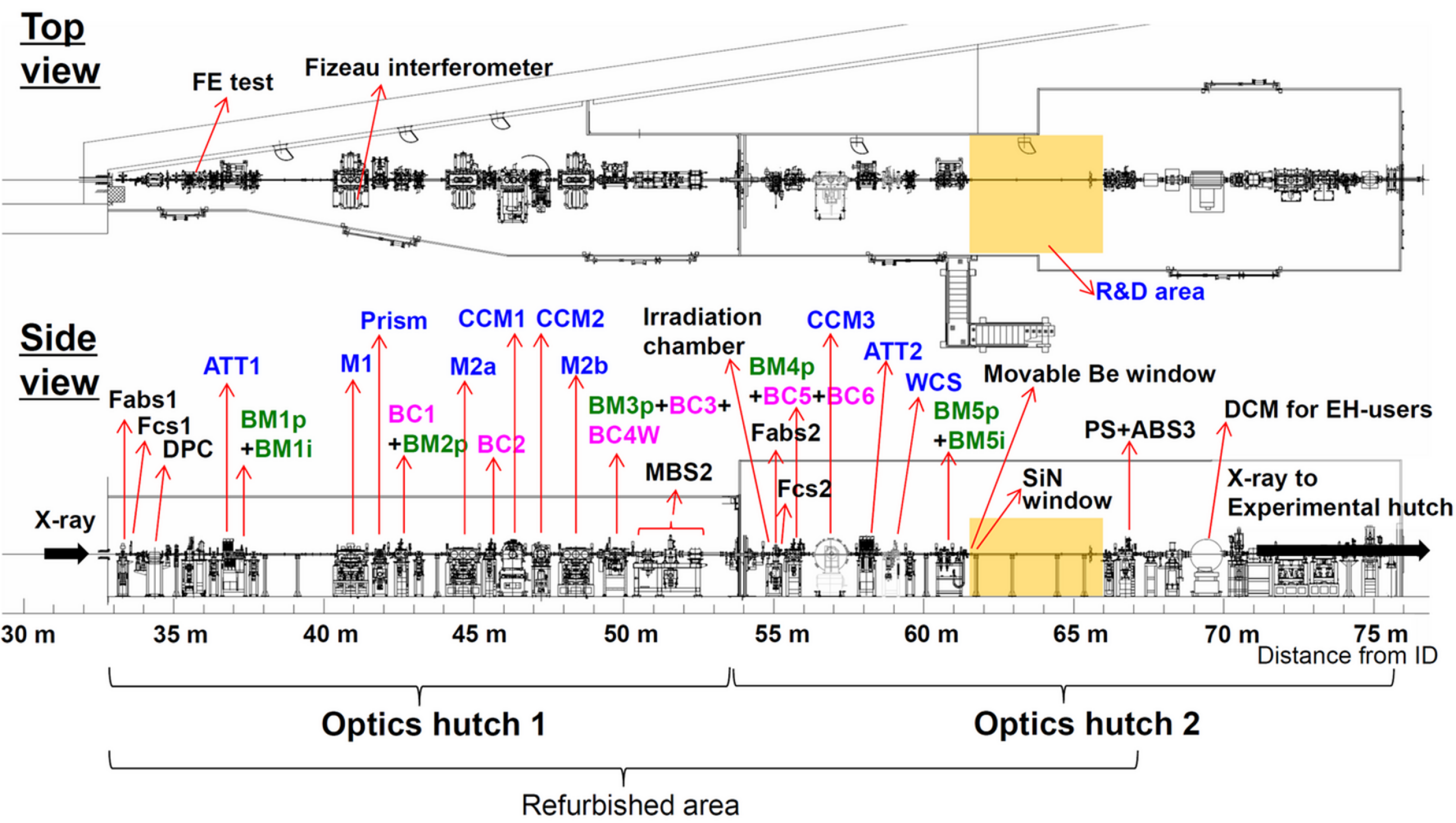
Configuration of beamline instruments. ATT: attenuator; M (M1, M2a, M2b): mirror; CCM: channel-cut monochromator; BM1p–BM5p: beam profile monitors; BM1i, BM5i: beam intensity monitors; BC: beam catcher; BC4W: BC with tungsten shutter; Fabs: fast absorber; Fcs: fast closing shutter; DPC: differential pump chamber; MBS2: main beam shutter 2; WCS: water cooling slits; PS: pink-beam stopper; ABS: absorber; DCM: double crystal monochromator.

**Figure 2:**
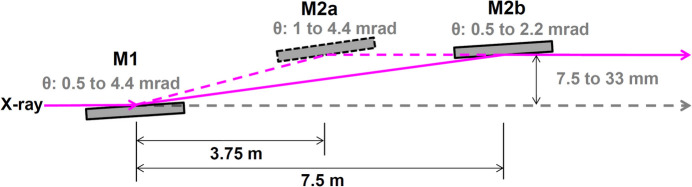
Optical layout of the double multilayer monochromator (DMM) with mirrors placed in OH1.

As illustrated in Fig. 1 ▸, most optical instruments in the first and second OHs (OH1 and OH2) were refurbished during the major modification in 2020. The main components are multilayer mirrors (M1, M2a and M2b), a harmonic separator prism, and a double channel-cut monochromator (DCCM) consisting of CCM1 and CCM2 chambers (Yumoto *et al.*, 2020 ▸), which deflect the beams vertically. The M1–M2a and M1–M2b pair have a DMM geometry, as shown in Fig. 2 ▸. In this study, we investigated the performance of the DMM with the M1–M2b pair, which extracts a higher-order harmonic at 100 keV and beyond. The M1–M2b pair, at an incident angle of 1.91 mrad, extracts a 100 keV beam with an offset of 29 mm. Higher-energy X-rays can be extracted from the DMM by choosing a smaller glancing angle.

X-ray instruments under high-heat-load conditions can be evaluated in these OHs, with a maximum power density of 266 W mm^−2^ at 36 m and 77 W mm^−2^ at 67 m from the source. A partial power of 420 W passes through the front-end (FE) slit (primary slit), with an aperture size of 0.56 mm (height) and 1.86 mm (width), at 29.1 m from the source. The attenuator (ATT) plates installed in the attenuator chambers ATT1 and ATT2 absorb low-energy components and control the heat load conditions for downstream instruments. A shutter with a water-cooled absorber [main beam shutter 2 (MBS2)] is installed at the end of OH1, allowing access to evaluation instruments in OH2. This stabilizes the DMM in OH1 by maintaining constant heat loads, enabling stable X-ray evaluations and analyses.

A 4 m × 3 m R&D area has been prepared in OH2 for the characterization of the optical components and the operation of pilot experiments with high-energy X-rays. Focusing mirror optics and a reflective beam expander (RBE) were evaluated in the R&D area, as described below.

### 2.3. Double multilayer monochromator

#### 2.3.1. Multilayer design, substrate and deposition

**Figure 3:**
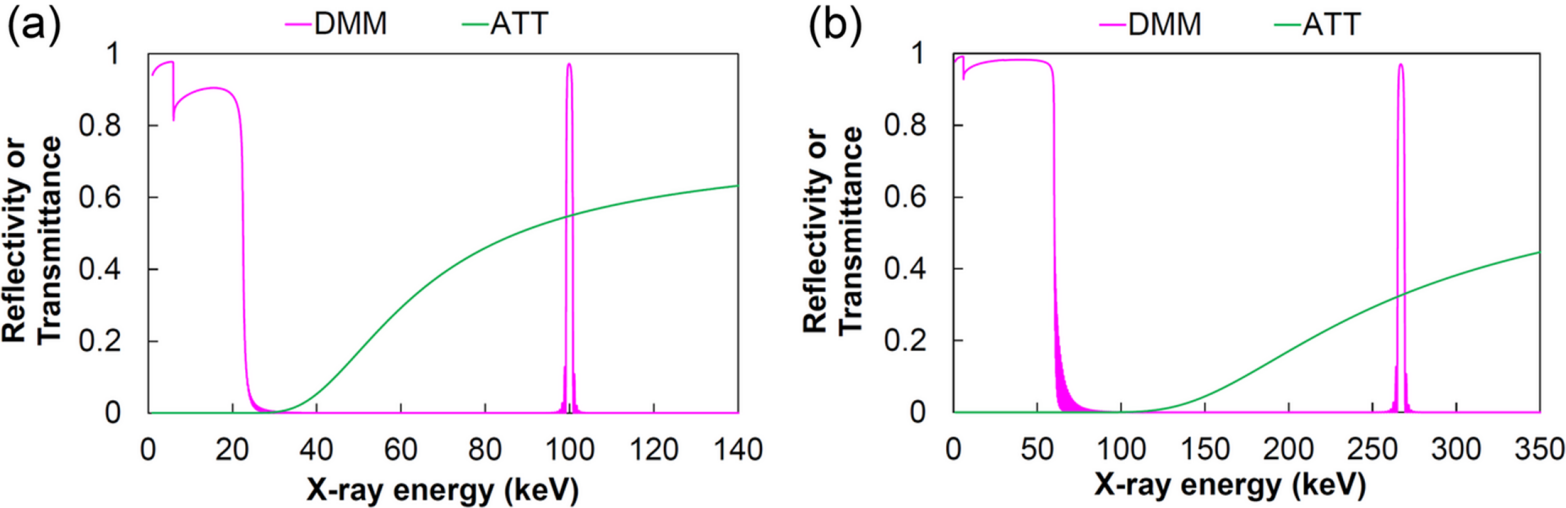
Calculated reflectivity of the DMM and transmittance of attenuators. The reflectivity curves of the DMM are shown with the first-order diffraction peaks at (*a*) 100 keV and (*b*) 267 keV.

The multilayer mirrors used for the DMM were designed to extract a high-flux beam with a 1% bandwidth at 100 keV. The design parameters are listed in Table 1 ▸. Fig. 3 ▸ shows the calculated reflectivity of the multilayers, as listed in Table 1 ▸, using the Parratt recursion formula (Parratt, 1954 ▸) and optical properties from the NIST database (Chantler *et al.*, 2009 ▸). The multilayer extracts 100 keV X-rays at an incident angle of 1.91 mrad as the first-order diffraction with a reflectivity of over 95%. ATTs are employed to suppress low energy components, which can be delivered by total reflection of the DMM. The typical attenuators and their respective thicknesses used in the calculations are as follows: (*a*) diamond (1.2 mm), SiC (1.4 mm), Si (8.0 mm) and Mo (0.1 mm); (*b*) diamond (1.2 mm), SiC (1.4 mm), Si (8.0 mm), Mo (2.0 mm) and W (0.6 mm). In addition, a maximum X-ray energy of 267 keV was evaluated at an incident angle of 0.72 mrad.

The substrates of the multilayer mirrors are made of single-crystal silicon. The substrate surfaces were finished with ultra-high precision by JTEC Corporation, with residual figure errors of 0.13 nm (M1) and 0.12 nm (M2b) [root-mean-square (RMS)] (evaluation length: 300 mm) and a surface roughness of 0.15 nm (RMS) (evaluation area: 140 µm × 110 µm), assessed by an optical interferometer. The multilayers were deposited using a custom-designed system at SPring-8.

Recent advances in multilayer coating technologies (Morawe *et al.*, 2007 ▸; Conley *et al.*, 2014 ▸; Ni *et al.*, 2019 ▸; Wang *et al.*, 2024 ▸) have enabled the fabrication of multilayers with key characteristics, including deposition over areas exceeding 1 m, more than 100 layers, high thickness uniformity, and periods below 2 nm (Singhapong *et al.*, 2024 ▸). To achieve high-precision thin-film optics, a dedicated deposition system was developed at SPring-8. This system employs DC magnetron sputtering, is equipped with four 2-inch-diameter targets, and supports coating substrates up to 600 mm in length. To ensure uniform deposition, the substrate is scanned across the sputtering sources. Film thickness is controlled by adjusting the scanning speed while keeping the sputtering rate constant (Morawe & Peffen, 2009 ▸).

#### 2.3.2. Cooling and alignment mechanisms

**Figure 4:**
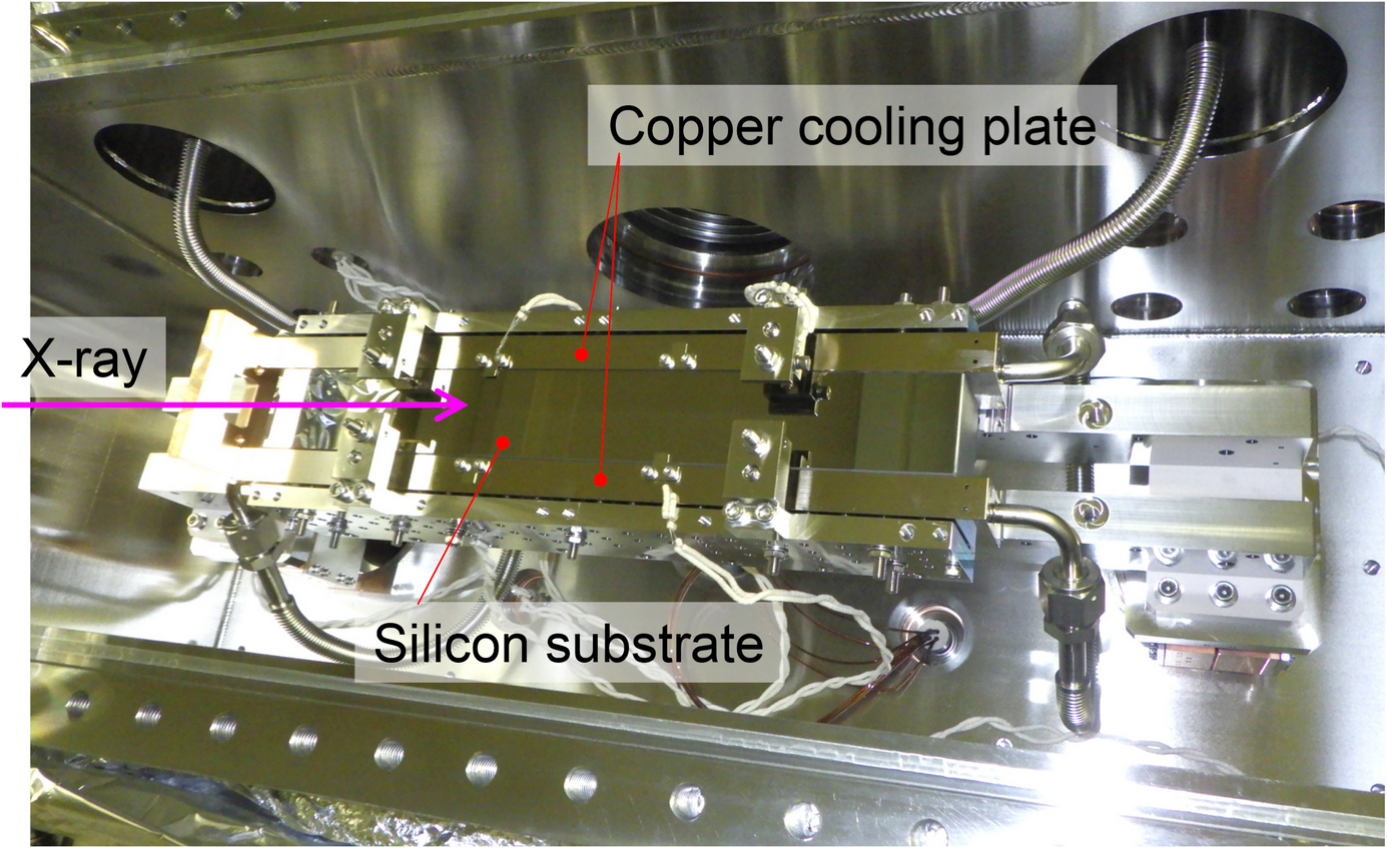
Photograph of the multilayer mirror (M1) and indirect cooling system. The silicon substrate is clamped between copper cooling plates.

The first multilayer mirror (M1) of the DMM is cooled cryogenically to minimize deformations caused by high-heat-load undulator radiation. Fig. 4 ▸ shows a photograph of M1 and its indirect cooling setup in an ultra-high vacuum chamber, operating under 3 × 10^−7^ Pa. The cooling system for M1 is designed to handle the maximum heat load from the undulator with a maximum power density below 1 W mm^−2^ on the M1 surface under grazing-incidence conditions.

The mirror and the cooling holder assembly in a vacuum are supported by two pairs of motorized stages, positioned outside the vacuum and connected through bellows. Each pair consists of horizontal and vertical translation stages. These four stages can adjust the incident angle, short-axis translation, vertical translation and in-plane rotation of the mirror. The same stage configuration is used for M1 and M2b. A heat shield plate is placed above the first mirror (M1) in a vacuum chamber to improve the thermal stability of the mirror by absorbing the inelastic scattering power from the mirror. The shield consists of copper plates connected to a water-cooling copper tube and tungsten plates to increase absorption of high-energy X-rays over several hundred keV. When the DMM extracts the 100 keV harmonic, the thermal load on M1 is 70 W after absorbing the low X-ray energy components using ATT1 [diamond (1.2 mm), SiC (1.4 mm), Si (8.0 mm) and Mo (0.1 mm)]. M2b is indirectly cooled with water due to the limited thermal load of below 10 W.

### 2.4. Other optical components

#### 2.4.1. Attenuators

**Figure 5:**
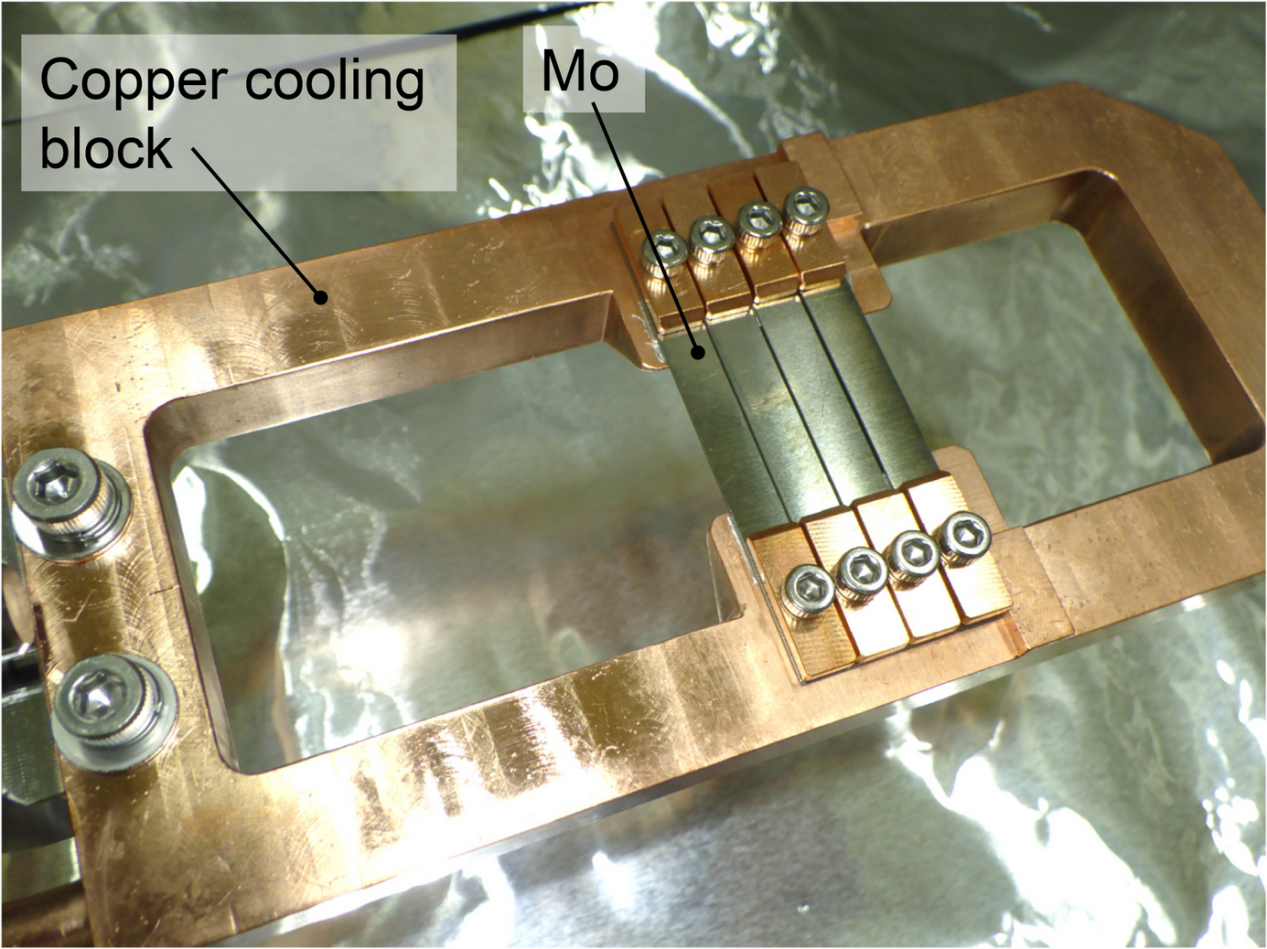
Photograph of axis number 5 (Mo) in the ATT2 assembly. Attenuation plates are attached to a copper block, which has a brazed copper tube on its back side to circulate cooling water. The attenuators consist of stacked molybdenum plates, each with a thickness of 0.4 mm, resulting in total thicknesses of 0.4, 0.8, 1.2 and 1.6 mm.

The attenuation plates in ATT1 are listed in Table 2 ▸, and are positioned with a motorized stage to select attenuator thickness. The attenuator materials are fixed on a water-cooled copper block to manage the heat load from the undulator source. By inserting axes number 1 to 6 in series, the maximum power of 420 W can be absorbed from the low-energy part, preventing thermal damage to the ATT materials. ATT2 has the same configuration as ATT1. Fig. 5 ▸ shows a photograph of the developed axis number 5 (Mo) in ATT2.

#### 2.4.2. Focusing mirror optics

X-ray focusing devices are crucial for improving spatial resolution and photon density. A sub-micrometre focusing device was developed, as reported in 2024 (Koyama *et al.*, 2024 ▸), utilizing laterally graded multilayer mirrors in Kirk­patrick–Baez geometry for the 100 keV pink beam. The focused beams at 100 keV have sizes of 0.25 µm (V) × 0.26 µm (H) and 0.32 µm (V) × 5.3 µm (H) [full width at half-maximum (FWHM)], with fluxes of 6 × 10^10^ and 1 × 10^12^ photons s^−1^, respectively.

#### 2.4.3. Reflective beam expander

**Figure 6:**
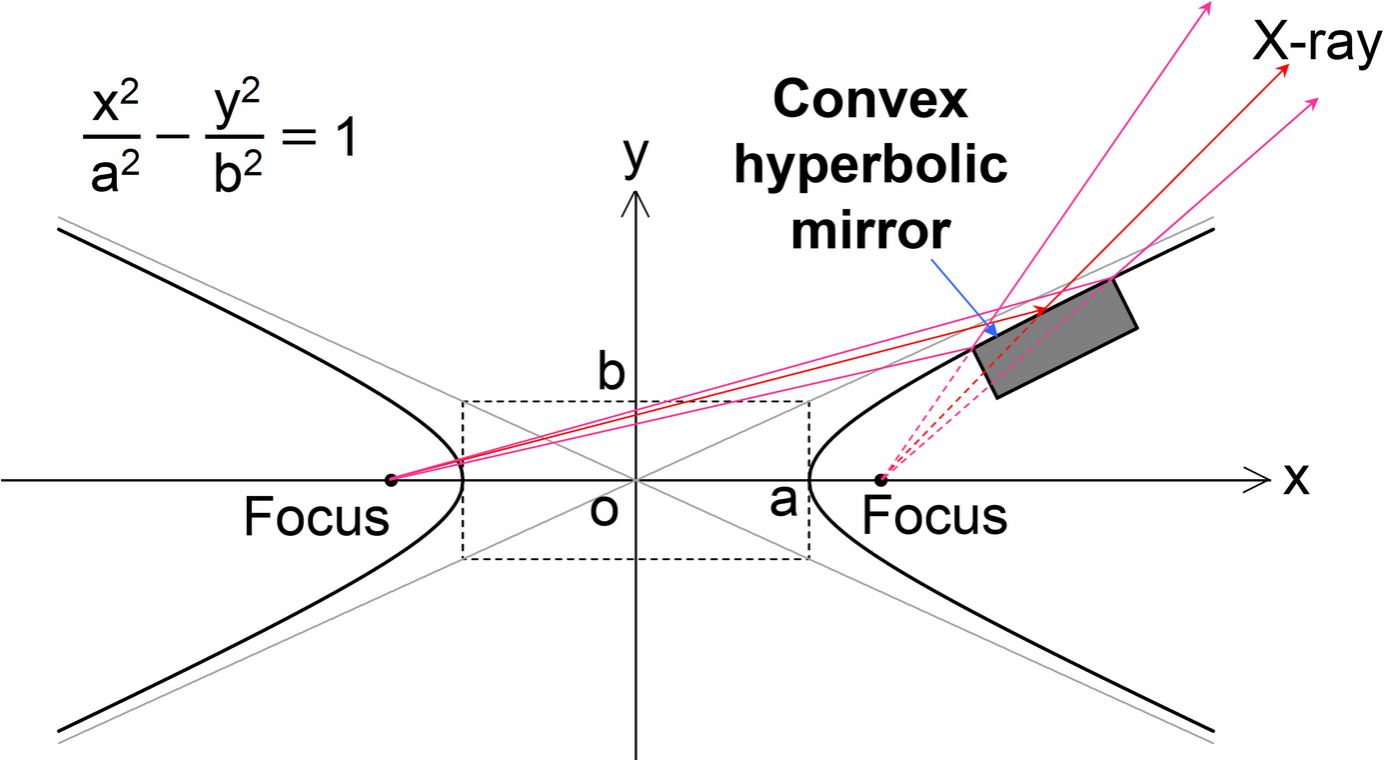
Geometry of the convex hyperbolic mirror.

For transmission imaging, it is preferred to achieve an illumination field of equivalent size vertically and horizontally at the sample position. However, in the R&D area, the 100 keV beam extends horizontally with a size of 0.7 mm (V) × 2.9 mm (H) (FWHM), as shown below. To address this issue, a one-dimensional (1D) RBE was designed to broaden the incident beam after being reflected by a convex hyperbolic mirror. As shown in Fig. 6 ▸, when a hyperbolic convex surface reflects light from the source (the farther focus), the beam diverges as if it originated from the near focus.

Table 3 ▸ shows the design parameters of the 1-D RBE for the 100 keV beam. A multilayer mirror achieves the same spatial acceptance with a shorter mirror length due to its larger glancing angle compared with a total reflection mirror. The average reflectivity of the 400 mm-long W/C multilayer mirror is calculated to be 87%. The multilayer materials were chosen for their wide incident-angle acceptance, preventing reflectivity loss due to misalignments, including mechanical instability. This mirror has a wide incident-angle acceptance of ±46 µrad (FWHM) at the center, compared with ±16 µrad (FWHM) for the DMM. A vertical beam size of 5.5 mm, larger than the horizontal beam size, is expected 3 m from the RBE. The multilayer was coated using magnetron sputtering in the in-house laboratory at SPring-8.

## 3. Performance

### 3.1. DMM performance at 100 keV

#### 3.1.1. Photon flux and spatial profile

The photon flux (defined here as the number of photons per second) of the 100 keV beam was measured with a Si PIN photodiode detector (S14537-320, Hamamatsu Photonics KK). The output current (0.476 mA) from the detector was converted into a photon count using equations (3) and (4) of Nariyama *et al.* (2004 ▸). The mass energy-absorption coefficient of silicon at 100 keV used in Nariyama *et al.*’s equation (4) was 0.04513 cm^2^ g^−1^, which was obtained from the NIST database (Chantler *et al.*, 2009 ▸). The evaluated photon flux was found to be 3.4 × 10^13^ photons s^−1^ at 1.0% bandwidth, while the estimated value was 7.7 × 10^13^ photons s^−1^ at 0.96% bandwidth.

**Figure 7:**
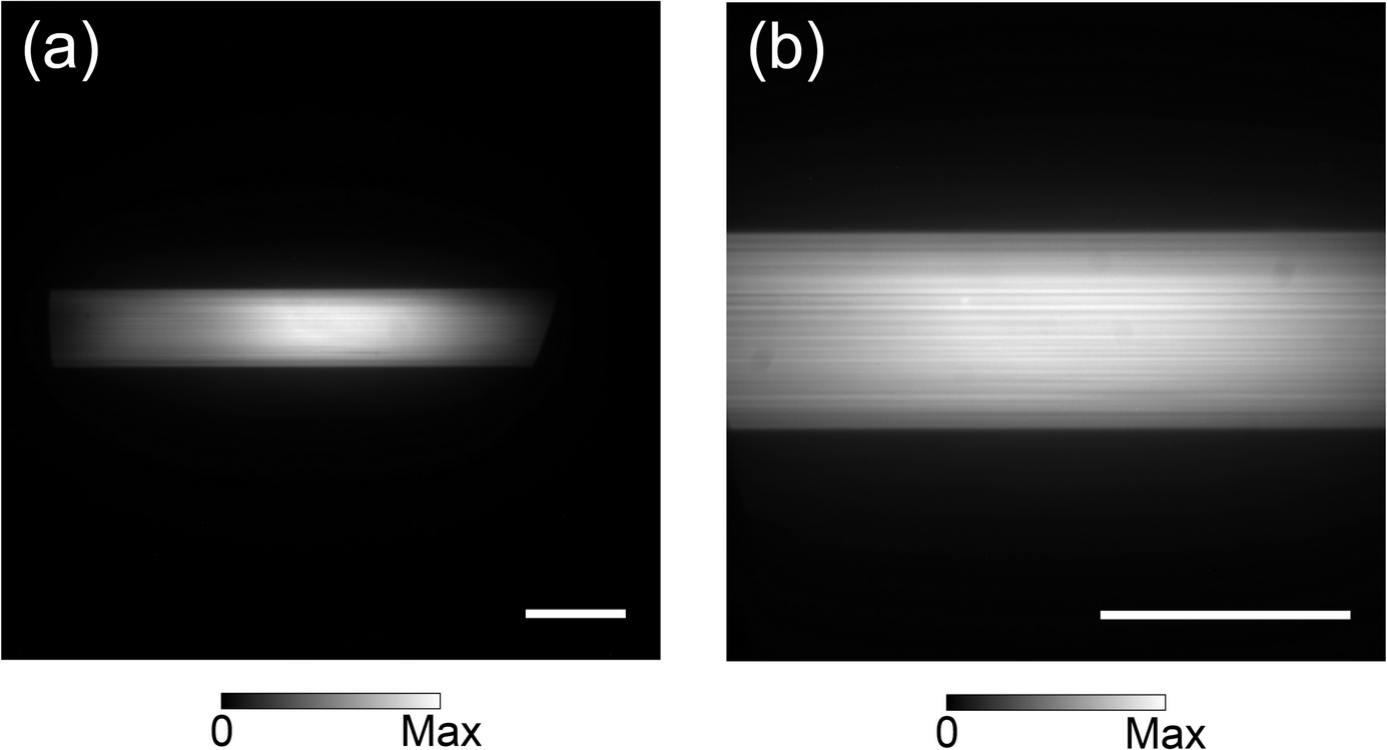
Beam profiles of a 100 keV beam. (*a*) An overall view of the beam. (*b*) An enlarged view of the beam’s central region. The scale bars represent 1 mm. The exposure times were (*a*) 30 ms and (*b*) 50 ms.

As shown in Fig. 7 ▸, we measured the spatial profiles of a 100 keV beam passing through the DMM using a DIFRAS X-ray camera with an indirect detection scheme (Kameshima *et al.*, 2019 ▸). The 2× and 5× detection systems, listed in Table 4 ▸, were used for the observations given in Figs. 7 ▸(*a*) and 7(*b*), respectively. The detection systems had a scintillator screen made of Ce-doped lutetium aluminium garnet (Lu_3_Al_5_O_12_) (LuAG:Ce) with a thickness of 10 µm and a CMOS camera (ORCA Flash4.0, Hamamatsu Photonics KK) with a resolution of 2048 × 2048 pixels.

The beam size at 1% bandwidth in Fig. 7 ▸(*a*) was evaluated to be 0.7 mm (V) and 2.9 mm (H) (FWHM), agreeing well with the calculation. The spatial profile of the beam does not show any notable thermal or clamping distortion of the DMM. Due to the longer spatial coherence in the vertical direction compared with the horizontal direction, the intensity variation in the vertical direction is easily influenced by surface errors of the mirrors. After removing the low-frequency component represented by a cubic curve, the measured intensity variation of the beam along the vertical center section of length 0.48 mm was 3.8% (standard deviation). According to wave-optical propagation calculations, this value can be attributed to the measured residual figure errors of the mirrors.

#### 3.1.2. Energy spectrum and reflectivity

**Figure 8:**
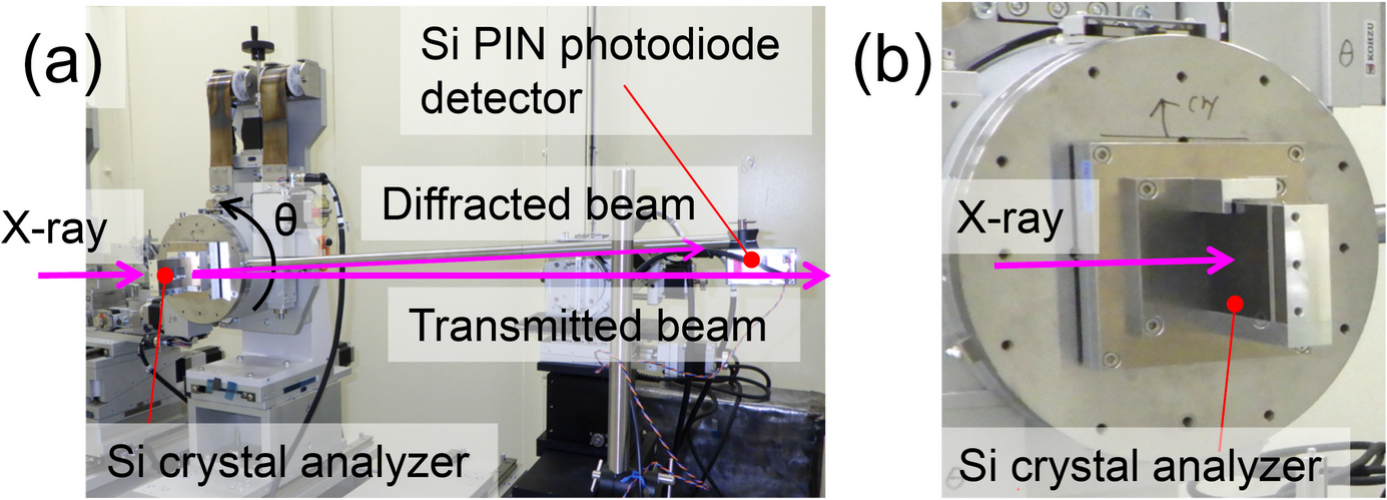
Experimental setup for energy spectrum measurement of the 100 keV beam. (*a*) Overall view, and (*b*) enlarged view of the Si crystal analyzer.

We evaluated the energy spectrum of the 100 keV beam. For the measurement, we used a Si crystal analyzer with 111 crystal planes, employing a θ–2θ scan method in the Laue diffraction geometry. Fig. 8 ▸ shows the experimental setup for the energy spectrum measurement. Diffraction intensity was measured with a Si PIN photodiode detector. The analyzer crystal has a thickness of 10 mm to prevent deformation caused by clamping.

**Figure 9:**
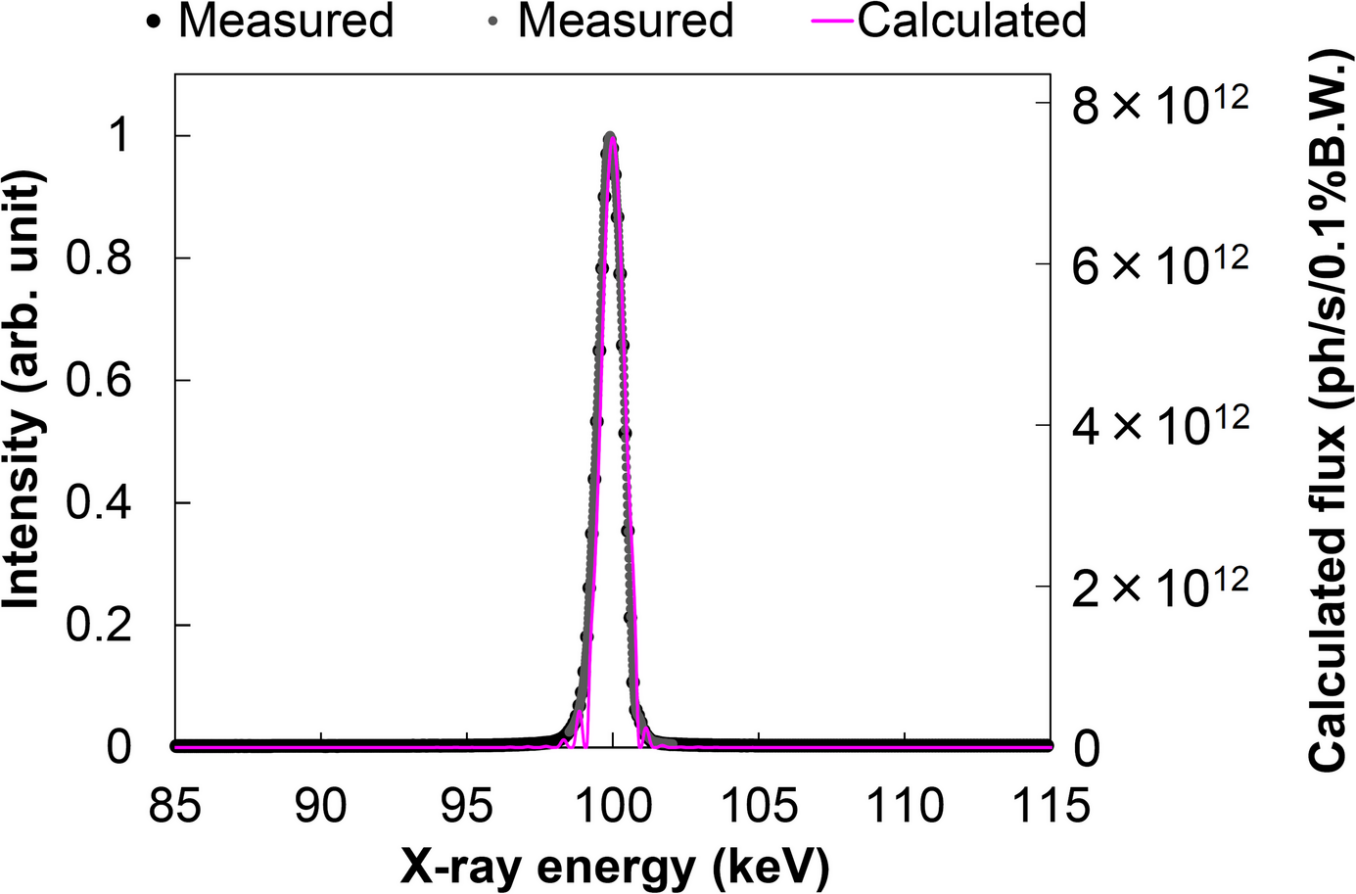
Measured and calculated spectra of the 100 keV beam extracted by the DMM. The vertical axis represents the measured results on the left and the calculated result on the right.

Fig. 9 ▸ shows the measured and calculated energy spectra of the 100 keV beam. The incident angle of the DMM was fine-tuned to extract the precise 100 keV energy at around the design incident angle of 1.91 mrad. The FE slit size of 0.41 mm (V) × 1.86 mm (H) and the ATT parameters used in the calculation of Fig. 3 ▸(*a*) were used for the evaluations. The undulator gap was fine-tuned to precisely produce 100 keV energy at the 19th harmonic peak. The evaluated spectra, which have an energy bandwidth of 1.0% with a Gaussian-like single peak, were consistent with the calculations.

**Figure 10:**
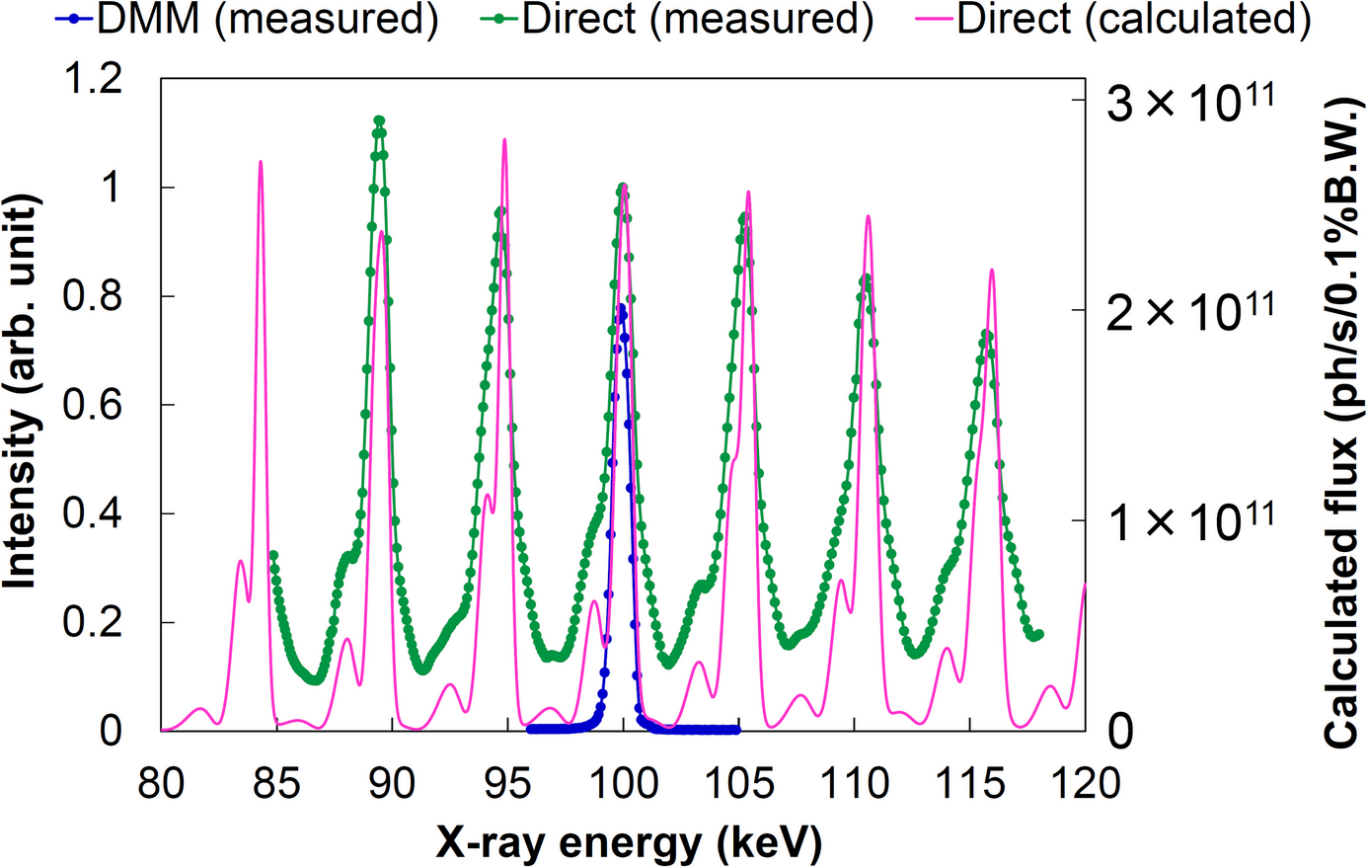
Spectra of the extracted 100 keV harmonic (measured) and undulator’s direct radiation (measured and calculated) obtained during evaluation of the DMM’s reflectivity. The vertical axis represents the measured results on the left and the calculated result on the right.

The spectrum of direct undulator radiation without monochromatization and that with the DMM were measured to evaluate the reflectivity of the DMM. For both measurements the X-ray intensity was attenuated sufficiently to reduce the thermal load for the crystal analyzer. Fig. 10 ▸ shows the measured and calculated spectra. The calculation is performed using the *SPECTRA* code (Tanaka, 2021 ▸). As designed, the 19th harmonic single peak at an X-ray energy of 100 keV was extracted by the DMM from multiple higher-order harmonic peaks. The reflectivity of the DMM was evaluated to be 78% according to the intensity ratio of the 100 keV peak, while the estimated value is 97% under a condition of multilayer roughness 0.3 nm (RMS).

The difference between the measured and estimated reflectivities primarily arises from Compton-scattering background originating from the Si crystal analyzer, particularly during the measurement of the undulator’s direct spectrum. Laboratory reflectivity measurements of multilayer coating samples indicate that interdiffusion/roughness (approximately 0.4 nm RMS) contributes less than 2% to the reflectivity loss. Therefore, the discrepancy between the estimated and measured photon flux can be attributed mainly to uncertainties in the estimation of the higher-order harmonic spectrum. As shown in Fig. 10 ▸, magnetic field imperfections in the undulator broaden the energy bandwidth of the higher-order harmonics, thereby reducing the photon flux at the 100 keV peak compared with the estimated value.

#### 3.1.3. Stability of intensity and position

**Figure 11:**
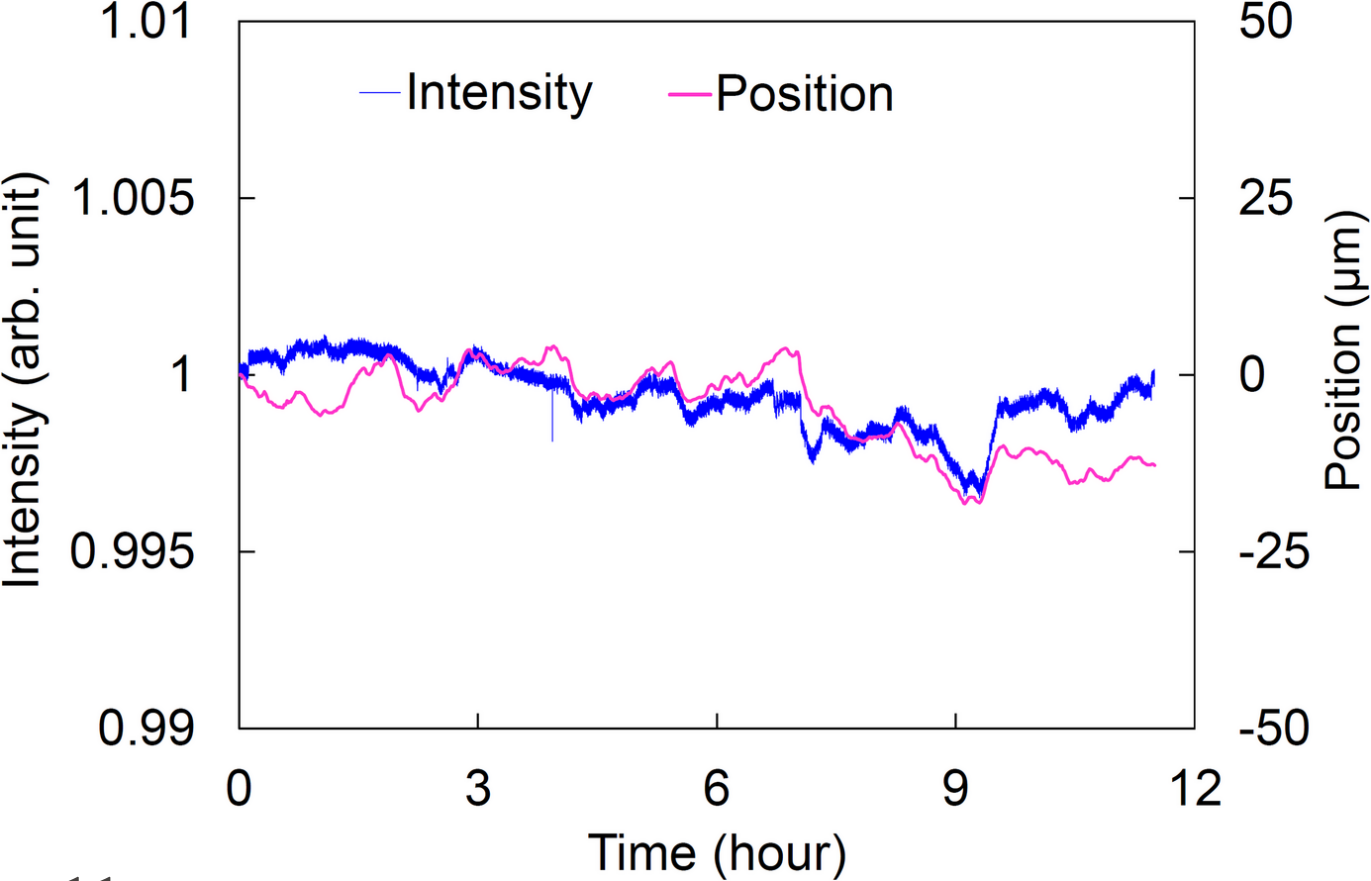
Time-dependent variations in intensity and position of the 100 keV beam.

We measured the time-dependent variations in the intensity and position of the 100 keV beam, as shown in Fig. 11 ▸. The intensity was measured using the Si PIN photodiode detector. Simultaneously, the spatial profiles of the beam were measured using the 5× detection system. Due to the beam’s horizontal elongation and the vertical deflection by the DMM, the vertical position stability was evaluated. Measurements began 24 h after the DMM was irradiated under the 100 keV conditions. The intensity fluctuated by 0.46% [peak-to-valley (PV)], and the beam position fluctuated by 22.3 µm (PV) over 11.5 h. Assuming the position change was due to the cryogenically cooled M1, the variation corresponds to an incident angle error of 0.5 µrad (PV). This angular fluctuation could cause the intensity fluctuation, as the beam position and intensity show a moderate correlation.

### 3.2. DMM performance above 100 keV

The DMM can select the higher photon energy by decreasing the incident angles of multilayers to satisfy the diffraction condition. The evaluation results are summarized in Table 5 ▸. The vertical size of the FE slit was adjusted to match the beam size at the spatial acceptance of the M2b for higher-energy X-rays. The horizontal size of the FE slit was fixed to be 1.86 mm. Attenuators were employed to absorb low-energy components. Although the photon flux at 267 keV decreased by two orders of magnitude compared with the 100 keV beam, a 1.2% bandwidth beam with a photon flux beyond 10^11^ photon s^−1^ is available.

## 4. Demonstration analysis

### 4.1. High speed imaging

We performed basic experimental studies on high-energy, high-flux X-ray transmission imaging at 100 keV. The image acquisition conditions are summarized in Table 6 ▸. A high-speed 2D movie and computed tomography (CT) were performed by acquiring X-ray absorption contrast images with a high-speed CMOS camera (FASTCAM SA-Z, Photron Ltd). This system had a resolution of 1024 × 1024 pixels and a pixel size of 4 µm on a LuAG:Ce scintillator screen with the 5× magnification objective.

**Figure 12:**
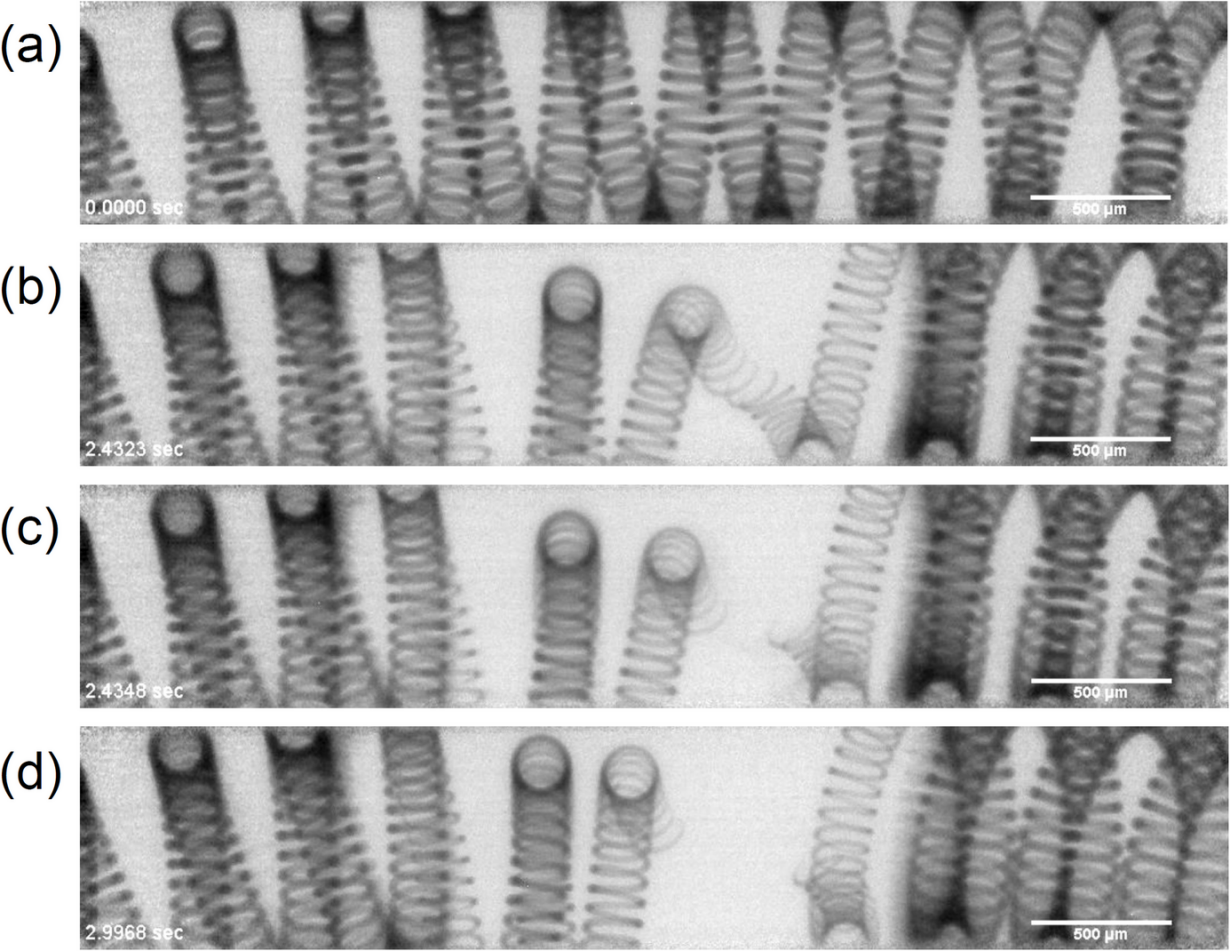
Frames of a high speed movie of vaporization of a tungsten filament by electrical resistance heating. Panels (*a*), (*b*), (*c*) and (*d*) show the filament at relative times of 0, 2.4323, 2.4348 and 2.9968 s, respectively. The scale bars represent 0.5 mm. The movie is shown in the supporting information.

Vaporization of a tungsten filament by electrical resistance heating in atmospheric conditions was observed at 10000 frames per second (fps), as shown in Fig. 12 ▸. The tungsten wire had a diameter of 0.05 mm. The transmission images showed the intensity distribution after dividing the original images by the incident beam without the sample. Due to the high-flux beam, the high-speed movie at 10000 fps had sufficient contrast and a high signal-to-noise ratio even at a high-penetration X-ray energy of 100 keV.

**Figure 13:**
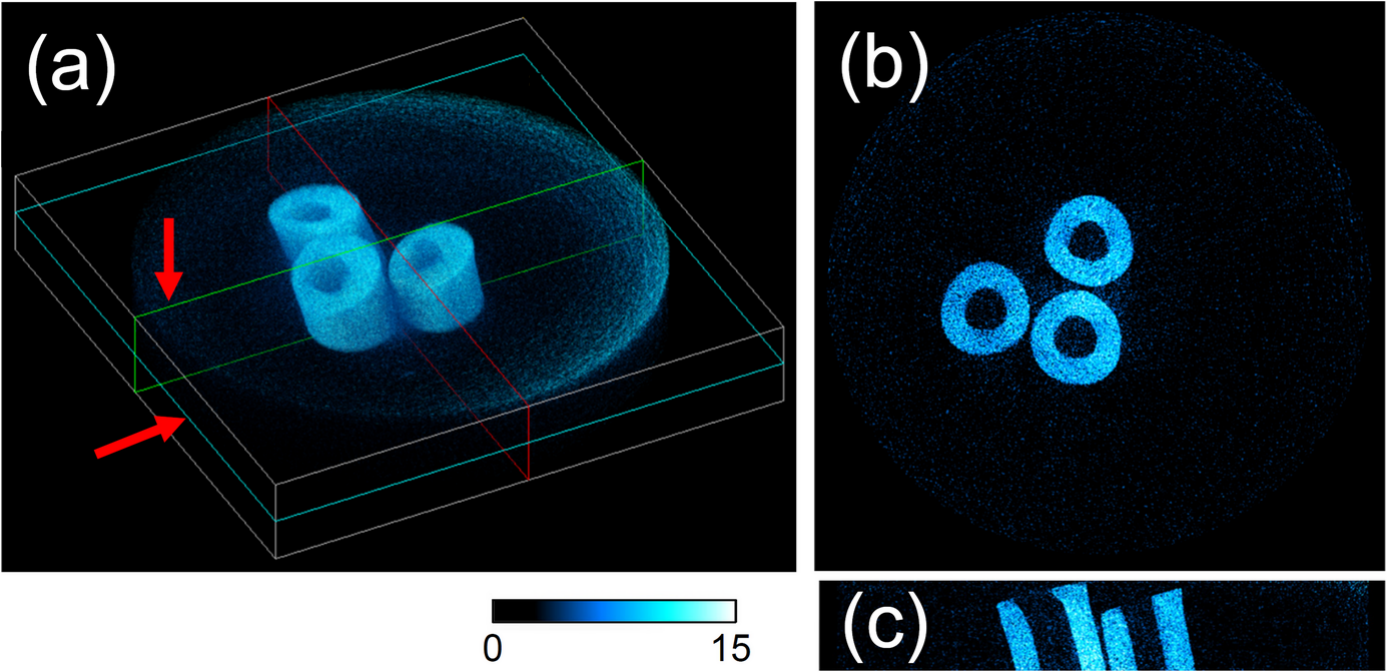
Computed tomography of lead-free solder with a data acquisition time of 2.45 ms. (*a*) Three-dimensional reconstruction. (*b*, *c*) Cross-sectional views of the light blue and green sections as indicated by the red arrows in (*a*). Mechanically cut edges are visible at the top of the reconstructed volume. The dimensions of the white box in (*a*) are 4.1 mm × 4.1 mm × 0.67 mm. The color bar represents the linear attenuation coefficient (cm^−1^) for (*b*) and (*c*).

The high-density lead-free solder (Sn 96.5%, Ag 3%, Cu 0.5%) was measured using high-speed CT. Fig. 13 ▸ shows tomograms of a three-wire solder sample with an external diameter of 0.6 mm. The reconstructed results clearly show through-holes along the center of the wires and cutting surfaces at a data acquisition time of 2.45 ms. The three-dimensional images were reconstructed using a conventional convolution-back projection algorithm with projection images at angles from 0 to 180°, as listed in Table 6 ▸.

**Figure 14:**
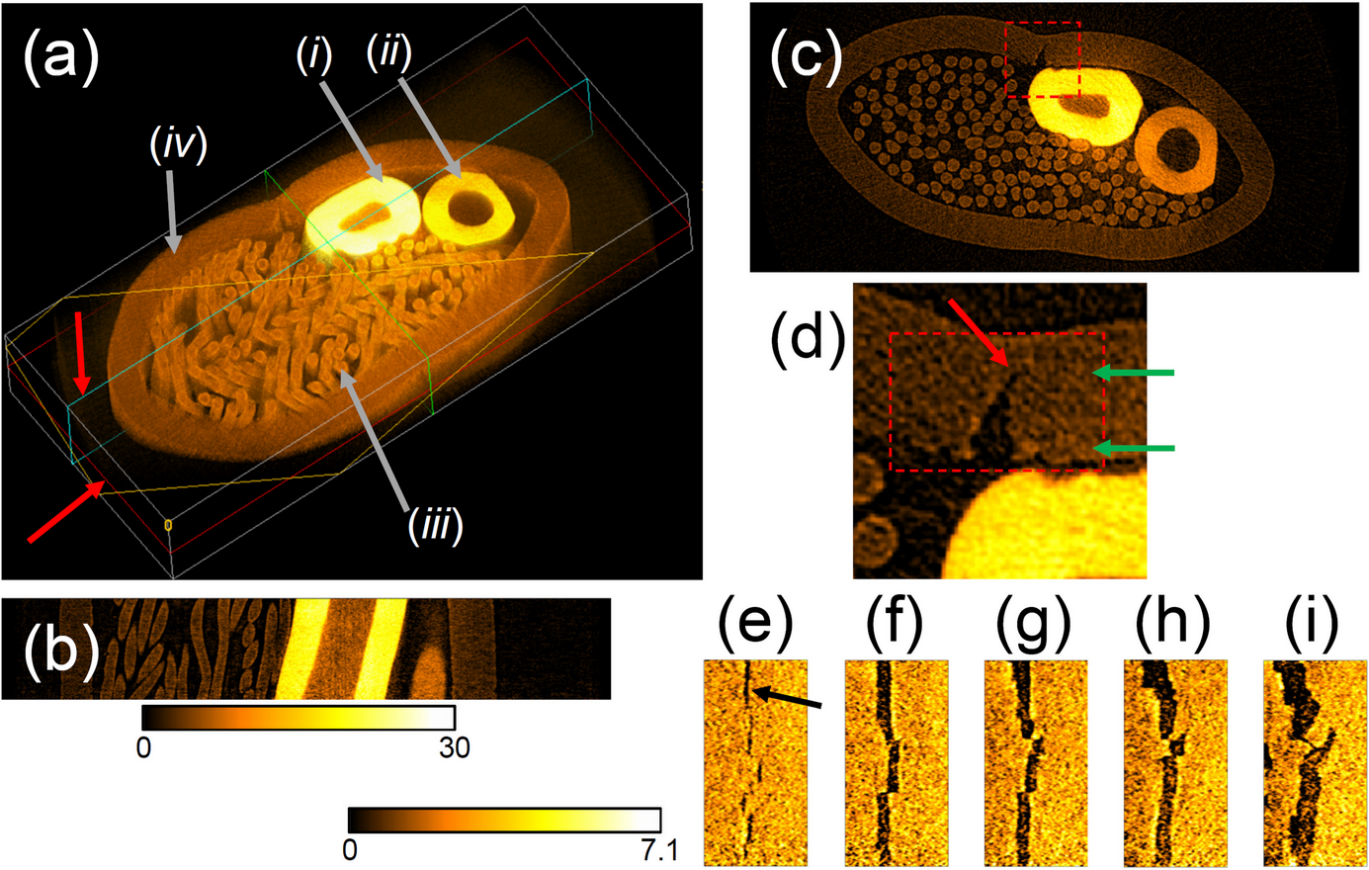
Computed tomography of a sample containing four metallic materials with a data acquisition time of 29.9 ms. (*a*) A three-dimensional reconstruction with a cross-sectional view along the yellow cross section. The white box in (*a*) measures 4.1 mm × 1.9 mm × 0.68 mm. (*b*, *c*) Cross-sectional views along the green and red sections indicated by the red arrows in (*a*). (*d*) Magnified view of the rectangular area indicated by the red dashed line in (*c*). The red arrow in (*d*) and black arrow in (*e*) indicate a crack. Panels (*e*)–(*i*) illustrate cross-sectional views of the rectangular area indicated by the red dashed line in (*d*) parallel to the green arrows. The dimensions of (*e*)–(*i*) are 336 µm × 676 µm. The distances between each cross-sectional view are 48 µm (*e*–*f*), 8 µm (*f*–*g*), 16 µm (*g*–*h*) and 20 µm (*h*–*i*). The color bars below (*b*) and to the left of (*e*) represent the linear attenuation coefficient (cm^−1^) for (*b*)–(*d*) and (*e*)–(*i*), respectively.

Fig. 14 ▸ provides another example of high-speed CT, visualizing a three-dimensional crack structure with a width as narrow as 10 µm in a metallic compound. The sample was prepared by inserting three materials into a stainless steel (SUS304) tube with an external (internal) diameter of 2.41 mm (1.99 mm), and then pressing to secure the materials. The inserted materials were: (i) wire solder (Sn 60%, Pb 40%) with an external diameter of 0.6 mm, (ii) lead-free wire solder (Sn 96.5%, Ag 3%, Cu 0.5%) with an external diameter of 0.6 mm, and (iii) braided copper wire (desoldering wick) with a diameter of 0.08 mm. Each wire solder had a through-hole filled with soldering flux along its center. The reconstructed results show complex internal structures of the high-density metal materials, with linear attenuation coefficients of 2.9 cm^−1^ for SUS304, 4.1 cm^−1^ for copper, 12.3 cm^−1^ for the lead-free solder and 27.4 cm^−1^ for the solder.

### 4.2. Enlarged-field imaging with beam expander

The 100 keV beam was enlarged vertically by the RBE after aligning the incident angle to maximize multilayer reflectivity. The vertical beam size increased to 6.1 mm. The measured reflectivity was 85%, compared with the calculated value of 87%.

**Figure 15:**
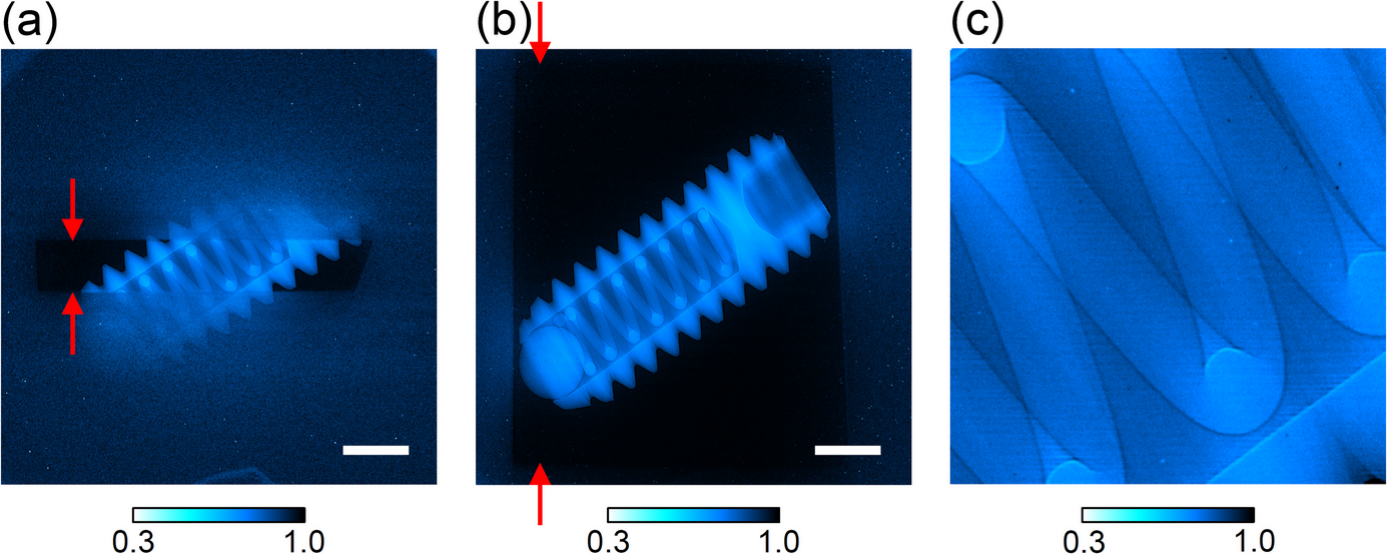
Evaluation result of RBE. Transmitted images of a sample ball plunger using (*a*) the standard beam and (*b*) the beam enlarged by the RBE. The vertical beam size, as indicated by the arrows, increased from 0.78 mm to 6.1 mm after the RBE enlargement. A magnified view of the 1 mm × 1 mm area at the center of (*b*) is shown in (*c*). The color bars represent transmittance ranging from 0.3 to 1.0. The scale bars represent 1 mm.

We measured the transmission image of a sample with the enlarged beam. The sample was a steel ball plunger, 5 mm in length, with an M2 thread (Part Number: FP-2, Nabeya Bi-tech Kaisha). The 2× detection system, positioned 3.3 m from the RBE center, measured the images as shown in Fig. 15 ▸, when the incident beam (*a*) was not enlarged and (*b*) was enlarged with the RBE. The sample was positioned 0.3 m in front of the detection system. The exposure times were 20 ms and 300 ms for Figs. 15 ▸(*a*) and 15(*b*), respectively. The RBE enables large-field imaging of a 5 mm square, enhancing the capability of high-energy, high-flux beam applications.

## 5. Discussion

We developed the beamline optics with DMM to utilize intense high-energy undulator radiation. We confirmed the high photon flux of 3.4 × 10^13^ photons s^−1^ at 100 keV, which is about 200 times higher than that from a conventional Si 511 DCM.

High-speed imaging at 100 keV is a powerful tool for observing high-speed phenomena, such as void generation and flow in molten iron during welding, which cannot be observed without high-penetrating X-rays. The high-speed tomography can create high-speed 3D movies to visualize the interior of high-density materials, such as crack propagation inside metals and solid-phase growth in molten metals. The 100 keV beam has been already applied for advanced X-ray analyses, such as transmission absorption imaging of aluminium alloy in spot welding (Iyota *et al.*, 2023 ▸; Funabiki *et al.*, 2024 ▸), Compton scattering imaging, 3D X-ray diffraction, X-ray diffraction measurement (Ohuchi *et al.*, 2024 ▸) and pair distribution function analysis (Fujita *et al.*, 2023 ▸; Kono *et al.*, 2024 ▸) under high-pressure.

**Figure 16:**
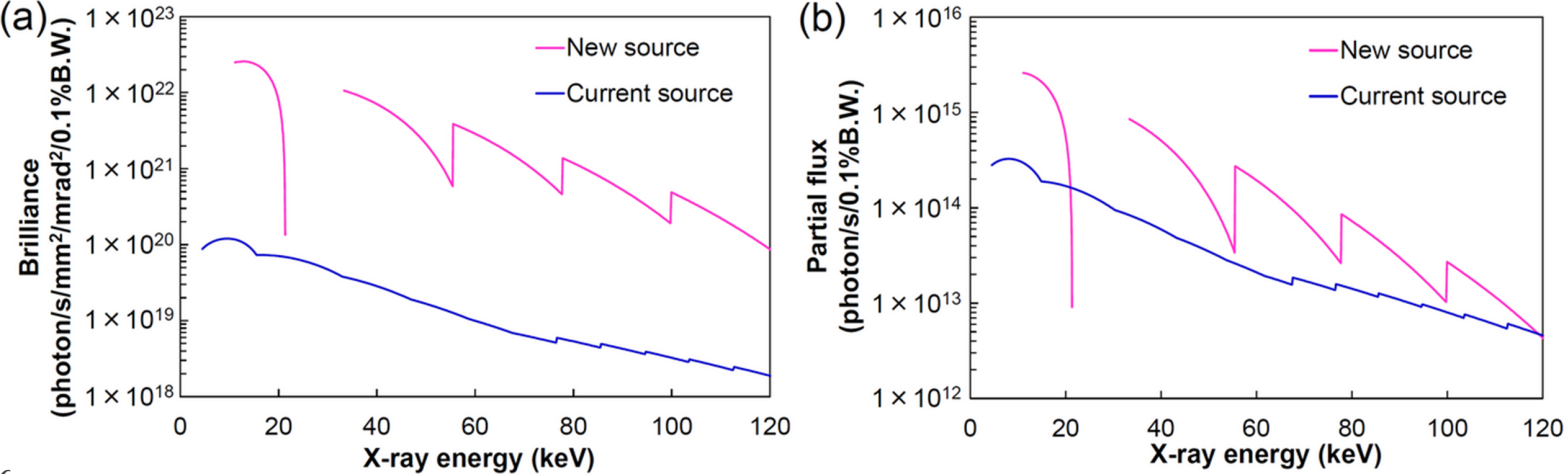
Estimated brilliance and partial flux of the SPring-8-II new source compared with that of the SPring-8 current source. (*a*) Brilliance and (*b*) partial flux are calculated using the source parameters listed in Table 7 ▸.

To achieve an extremely brilliant source using an ultra-low-emittance storage ring, the SPring-8 upgrade project (SPring-8-II) (Tanaka *et al.*, 2024 ▸) plans to change the beam energy of the storage electrons from 8 GeV to 6 GeV. However, a decrease in beam energy will result in a shift of the undulator radiation spectrum to the lower energy side due to the reduction in γ, as shown in equation (1). To increase the flux, the stored current will be increased from 100 mA to 200 mA. Furthermore, short-period cryogenic undulators (Hara *et al.*, 2004 ▸) can produce high-flux X-rays in the high-energy regions as they can achieve large *K* values with short undulator periods. Specifically, the source parameters in Table 7 ▸ provide exceeding a 100-fold increase in brilliance and exceeding a three-fold increase in partial flux at 100 keV compared with the current source. This is achieved with the ninth harmonic of undulator radiation, as shown in Fig. 16 ▸. The partial flux was calculated at a FE slit aperture of 0.5 mm × 0.5 mm.

## Supplementary Material

Supporting video. DOI: 10.1107/S1600577525006903/yn5123sup1.avi

## Figures and Tables

**Table 1:** Optical parameters of the multilayer mirrors

Substrate	Material	Single-crystal silicon
	Dimensions	400 mm (X-ray axis direction) × 50 mm (width) × 50 mm (thickness)
	Shape	Plane

Multilayer	Material	Cr/C
	Number of periods	150
	Period (*d*-spacing)	3.33 nm
	Gamma (Cr-layer thickness/period)	0.5
	Coated area	300 mm (X-ray axis direction)
	Roughness, interdiffusion	0.3 nm (RMS) (assumed)

DMM	Grazing incident angle	1.91 mrad (100 keV)
		0.72 mrad (267 keV)
	Spatial acceptance	570 µm (vertical) (100 keV)
		210 µm (vertical) (267 keV)
	Energy bandwidth (Δ*E*/*E*)	1.6% (100 keV)
		1.6% (267 keV)

**Table 2:** Design parameters of attenuators

Axis number		No. 1	No. 2	No. 3	No. 4	No. 5	No. 6
Material		Diamond single crystal	SiC single crystal	Silicon single crystal	Molybdenum metal (polycrystal)	Molybdenum metal (polycrystal)	Tungsten metal (polycrystal)
Effective thickness (mm)	Position 1	0.3	0.35	2.0	0.1	0.4	0.3
	Position 2	0.8	0.70	4.0	0.2	0.8	0.6
	Position 3	1.0	1.05	6.0	0.3	1.2	0.9
	Position 4	1.2	1.40	8.0	0.4	1.6	1.2
Surface quality (polish)		Mirror polished	Mirror polished	Mirror polished	Not polished	Not polished	Not polished
Incident angle		Normal incidence	Normal incidence	45° incidence	Normal incidence	Normal incidence	Normal incidence
Material superposition		None	Layered structure	Layered structure	Layered structure	Layered structure	Layered structure

**Table 3:** Design parameters of one-dimensional reflective beam expander (1D-RBE)

Surface profile	Hyperbolic convex
Substrate material	Single-crystal silicon
Mirror substrate size	400 mm × 50 mm × 50 mm (thickness)
Focal length	62 m (light source to mirror center)
	0.5 m (virtual focus to mirror center)
Semimajor axis (*a*)	30750 mm
Semiminor axis (*b*)	11.13552 mm
Glancing angle on optical axis	2.0 mrad
Surface coating	(W/C)_50_ laterally graded multilayer
Effective mirror length	390 mm
Spatial acceptance	780 µm
Calculated reflectivity	87%
Beam size at 3 m from mirror center	5.5 mm

**Table 4:** Parameters of the image detection system

	2× detection system	5× detection system
Magnification of objective	2×	5×
Numerical aperture of objective	0.055	0.14
Pixel size on scintillator screen	3.25 µm	1.30 µm
Field of view	6.65 mm × 6.65 mm	2.66 mm × 2.66 mm

**Table 5:** Evaluation results of DMM

Energy (keV)	Incident angle of multilayer mirror (mrad)	Δ*E*/*E* (%)	FE slit size: vertical (mm)	Beam size: vertical (mm) (FWHM)	Photon flux (photon s^−1^)	*K* value of undulator	Transmittance of attenuator
100	1.91	1.0	0.41	0.7	3.4 × 10^13^	2.28	0.55
130	1.47	1.2	0.27	0.6	1.2 × 10^13^	2.45	0.55
200	0.96	1.2	0.18	0.3	1.6 × 10^12^	2.53	0.52
267	0.72	1.2	0.18	0.3	1.3 × 10^11^	2.54	0.33

**Table 6:** Image acquisition conditions for high-speed imaging

Object	Vaporization of tungsten filament (Fig. 12 ▸)	Lead-free solder (Fig. 13 ▸)	Metallic compound (Fig. 14 ▸)
2D or 3D	2D	3D	3D
Frame rate	10000 fps	40000 fps	20000 fps
Shutter speed	98 µs	23 µs	48 µs
Scintillator thickness	1 mm	0.3 mm	0.3 mm
Rotation speed of sample	–	12300 rpm	1000 rpm
Number of projection images	–	98	598
Pixel or voxel size of image	4 µm×4 µm	4 µm×4 µm×4 µm	4 µm×4 µm×4 µm
Data acquisition time for CT	–	2.45 ms	29.9 ms

**Table 7:** Parameters of light sources

		New source SPring-8-II	Current source SPring-8
Beam energy (GeV)		6	8
Emittance (pm rad)		50	2400
Stored current (mA)		200	100
Natural energy spread (%)		0.098	0.109
Beta functions (β*x*, β*y*) (m) at undulator		(8.2, 2.8)	(31.2, 5.0)
Coupling ratio (%)		10	0.2
FE slit size (V × H) (mm)		0.5 × 0.5	0.5 × 0.5
Undulator	Maximum *K* value	1.36	2.28
	Magnetic period (mm)	16	32
	Number of periods	200	93
Brilliance at 100 keV [photon s^−1^ mm^−2^ mrad^−2^ (0.1% bandwidth)^−1^]		5 × 10^20^	3 × 10^18^
Partial flux at 100 keV [photon s^−1^ (0.1%bandwidth)^−1^]		3 × 10^13^	8 × 10^12^

## Data Availability

The data that support the findings of this study are available from the corresponding author on reasonable request.
